# Genome-wide association study of kernel colour traits and mining of elite alleles from the major loci in maize

**DOI:** 10.1186/s12870-023-04662-5

**Published:** 2024-01-03

**Authors:** Weiwei Chen, Fangqing Cui, Hang Zhu, Xiangbo Zhang, Siqi Lu, Chuanli Lu, Hailong Chang, Lina Fan, Huanzhang Lin, Junteng Fang, Yuxing An, Xuhui Li, Yongwen Qi

**Affiliations:** 1 Institute of Nanfan & Seed Industry, Guangdong Academy of Science, Guangzhou, 510316 Guangdong China; 2 College of Agriculture and Biology, Zhongkai University of Agriculture and Engineering, Guangzhou, 510325 Guangdong China; 3 College of Agriculture, Yangtze University, Jingzhou, 434025 Hubei China

**Keywords:** Maize, Kernel colour, Genome-wide association study, Candidate gene, Elite allele

## Abstract

**Background:**

Maize kernel colour is an important index for evaluating maize quality and value and mainly entails two natural pigments, carotenoids and anthocyanins. To analyse the genetic mechanism of maize kernel colour and mine single nucleotide polymorphisms (SNPs) related to kernel colour traits, an association panel including 244 superior maize inbred lines was used to measure and analyse the six traits related to kernel colour in two environments and was then combined with the about 3 million SNPs covering the whole maize genome in this study. Two models (Q + K, PCA + K) were used for genome-wide association analysis (GWAS) of kernel colour traits.

**Results:**

We identified 1029QTLs, and two SNPs contained in those QTLs were located in coding regions of *Y1* and *R1* respectively, two known genes that regulate kernel colour. Fourteen QTLs which contain 19 SNPs were within 200 kb interval of the genes involved in the regulation of kernel colour. 13 high-confidence SNPs repeatedly detected for specific traits, and AA genotypes of rs1_40605594 and rs5_2392770 were the most popular alleles appeared in inbred lines with higher levels. By searching the confident interval of the 13 high-confidence SNPs, a total of 95 candidate genes were identified.

**Conclusions:**

The genetic loci and candidate genes of maize kernel colour provided in this study will be useful for uncovering the genetic mechanism of maize kernel colour, gene cloning in the future. Furthermore, the identified elite alleles can be used to molecular marker-assisted selection of kernel colour traits.

**Supplementary Information:**

The online version contains supplementary material available at 10.1186/s12870-023-04662-5.

## Introduction

Maize (*Zea mays L*.) is one of the world's three major staple foods and is an important feed and industrial crop [1]. Kernel colour is the main index used to evaluate its commodity quality and value. The pigment in maize grain has a high nutritional value, and maize kernels have healthy beneficial functions [2, 3]. Maize kernel pigments mainly include two kinds of natural pigments: carotenoids and anthocyanins [4, 5]. Among them, carotenoids serve a variety of functions, including antioxidative, immune regulation, anticancer and antiaging functions; they are natural antioxidants and colorants and are also the major source of vitamin A in animals [6, 7]. Anthocyanins are flavonoid pigments that also have good antioxidant, free radical scavenging, antitumour, antiaging and skin beautifying effects and have many medical applications [2, 4, 8, 9].

Plants can produce carotenoids, instead of vitamin A, while carotenoids can be converted into vitamin A with physiological activity in the body, a process that can provide necessary vitamin A [6]. Not eating enough vitamin A can result in vitamin A deficiency (VAD), characterized by night blindness, anaemia, impaired immunity, and even death [10]. Instantly supplying vitamin A or improving the diet can reverse the effects of VAD, but chronic lack of vitamin A can lead to irreversible effects. Maize, a major food crop worldwide, is the main focus of carotenoid biofortification [11, 12].

Maize kernel colour is related to the key gene in carotenoid biosynthesis [13]. *Y1,* encoding phytoene synthase, is the first committed step in carotenoid biosynthesis [14]. *Y1* results in an orange kernel colour, and *y8* transforms the endosperm of the *Y1* background into light yellow. *Wc* also converts the yellow endosperm caused by *Y1* into white [15, 16]. Lycopene is the branch point of carotenoid biosynthesis and is regulated by lycopene β-cyclase (LCYB) and lycopene ε-cyclase (LCYE) [17, 18]. Downregulated expression of *LcyE* can cause a greater accumulation of β-branch carotenoids than α-branch carotenoids, and *LcyE* is located on chr8 and affects kernel colour [19, 20]. *Ps1* and *Vp5* are involved in core carotenoid biosynthesis, and *Vp5* is a white kernel mutant that lacks ABA [21]. *Vp14* is involved in the cleavage of carotenoids and is also related to the accumulation of kernel colour [16, 22].As inprevious study, two major QTLs were mapped on chr6 and chr9, and one of them was a *Y1* gene that controls the coloration of yellow and white kernel [23]. Brenda et al. (2019) identified the known genes *Y1* and *DXS2* by association analysis and explored the relationship between *DXS3*, *DMES1*, *LCYE*, *EP1* and the formation of kernel colour [13]. Chandler performed the visual scored by kernel colour for GWAS, and 11 QTLs were identified, *y1*, *lcyE*, *zep1*, and *ccd1* were associated with common QTL [22].

Anthocyanins are water-soluble flavonoids in plants that give plants a wide variety of colours and are robust against adversity experienced by plants [9]. Anthocyanins have an abundance of nutritional and medicinal abilies that enable all kinds of good things for human health [2, 24, 25]. Anthocyanins exist mostly in the aleurone layer of maize kernels, and purple corn is particularly rich in anthocyanins [4, 26]. The anthocyanin biosynthetic pathway involves many structural genes and the regulatory factors of these structural genes [4]. Structural genes encoding chalcone synthase (CHS) and chalcone isomerase (CHI) are key enzymes upstream of the anthocyanin biosynthetic pathway, and their expression levels are positively associated with anthocyanin content [27, 28]. The typical anthocyanin regulatory complex MBW consists of an R2R3-MYB protein, a basic helix-loop-helix (bHLH) protein, and a WD-repeat (WDR) domain protein [29, 30]. Studies of the anthocyanin biosynthetic pathway in maize show that the formation of purple aleurone is controlled by multiple genes, including *coloured aleurone 1* (*C1*, MYB), *coloured 1* (*R1*, bHLH), *and pale aleurone colour 1* (*Pac1*, WDR) [31–34]. *Intensifier1* (*In1*), with similarity to *R1,* encodes a bHLH-like recessive intensifier that increases the accumulation of anthocyanins in starch [32]. *Booster1* (*B1*) and *plant color1* (*Pl1*) are regulators of bHLH and MYB, respectively, and are related to the regulation of plant tissues [35].

Mature anthocyanins are transported into vacuoles for storage [4, 36]. According to reports, glutathione *S*-transferases (GSTs) catalyse conjugation between γ-Glu-Cys-Gly (GSH) and cyanidin-3-glucoside (C_3_G) or transport anthocyanins into the vacuolar membrane as carriers [37, 38]. Multidrug resistance-associated protein (mrp), an ABC transporter located on vacuolar membranes, can recognize anthocyanins and transport them across the membrane to the vacuole. Multidrug-resistant resistance-associated proteins located on the vacuole membrane recognize anthocyanin glycosides and transport them into the vacuole across the membrane [39, 40]. Chatham and Juvik performed association mapping in purple corn populations, Major QTLs for anthocyanin type were identified: *Pr1*, *R1* and the plant color-associated MYB, *Pl1* [4]. The GWAS of colour variation in rice, twenty-six loci were identified, and at least three candidates involved in the flavonoid metabolic pathway [41]. In durum wheat, The genetic mapping identified 4 QTLs disclosed the candidate genes *Pp-A3*, *Pp-B1*, *R-A1*, *R-B*, bHLH (*Myc-1*) and MYB (*Mpc1*, *Myb10*) [42].

The formation of maize kernel colour is regulated by a series of genes, and it is easily observed and closely related to nutritional quality. In this study, we used natural populations including 244 maize elite inbred lines and used the RGB colour model and visual classification (level) to evaluate the kernel colour, which served as the phenotype. To perform genetic analysis and mine the important genes, we then conducted a genome-wide association study on kernel colour with 3 million SNP markers covering the whole genome of maize. This will help in the deep examination of maize nutritional function and will provide support for the development of the maize industry from the quality of appearance.

## Results

### Phenotypic data statistical analysis

**Fig. 1:**
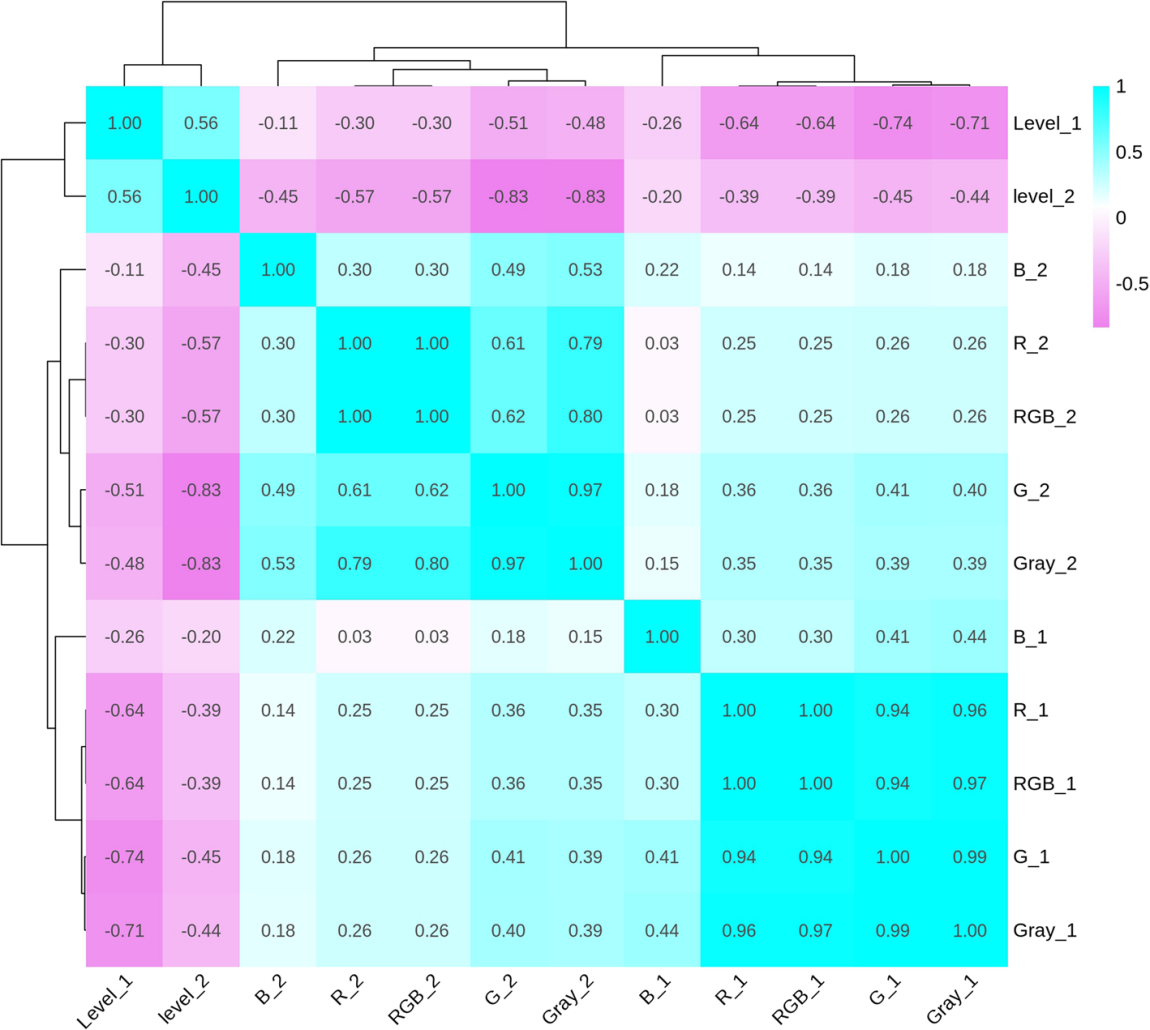
The correlations analysis among the 6 kernel colour traits in two environments. _1: wengyuan experimental station (2020); _2: guangzhou experimental station (2021); The number in the rectangle is the correlation coefficient, Purple indicates negative correlation, Cyan indicates positive correlation, the darker the color the higher the correlation

A statistical analysis was conducted for 6 kernel colour traits of the associated populations in two environments. The results showed that the variation range of the 6 traits was large, and the variation coefficients (CV) were all greater than 10% (Table 1). The absolute values of skewness and kurtosis were lower (Table 1). The maize kernel colour was divided into 7 grades by visual scoring, the darker kernel colour with the higher grades. Kernel colour had a rich genetic diversity in this population (Table 1). Combined with the frequency histogram, these 6 traits satisfy to the heredity of quantitative traits (Figure S1). The correlation coefficient between level 1 and level 2 was 0.56 (*P* < 0.001) (Fig. 1). There was a negative correlation between level and other colour traits, and a positive correlation between B and R, G, B, and RGB; the correlation coefficient was 0.3–0.53 across the two environments (Fig. 1). The correlation between R, G, B, and RGB was 0.61–0.97, showing a significant positive correlation (Fig. 1). The heritabilities of colour traits were 0.43, 0.59, 0.78, 0.6, 0.43 and 0.8, respectively (Fig. 1), which showed that the colour traits had higher heritability. Correlation analysis showed that the colour traits were positively correlated between the two environments.

**Table 1:** Descriptive statistics for kernel colour traits in two environments

Traits	En	Mean	Rang	SD	CV (%)	Kurtosis	Skewness	h^2^
R	E1	193.85	74.91–249.77	26.24	13.54	1.09	-0.53	0.43
	E2	182.51	20–240	25.86	14.17	12.51	-1.95	
G	E1	160.11	45.20–225.09	27.27	17.03	0.84	-0.47	0.59
	E2	123.72	10–195	29.98	24.23	1.03	-0.05	
B	E1	68.2	36.47–181.19	24.34	35.69	8.7	2.72	0.78
	E2	35.5	10–70	15.13	42.62	-0.77	0.2	
Gray	E1	159.72	53.89–220.52	25.03	15.67	0.9	-0.35	0.6
	E2	131.24	60.38–193.52	24.09	18.36	0.13	0.2	
RGB	E1	1.27E + 07	4.92E + 06–1.64E + 07	1.72E + 06	13.54	1.09	-0.53	0.43
	E2	1.20E + 07	1.35E + 06–1.58E + 07	1.70E + 06	14.17	12.3	-1.92	
Level	E1	3.74	1—7	1.13	30.46	0.75	0.21	0.8
	E2	4.26	1—6	0.87	20.42	0.45	-0.33	

*En* Environment, *E1* Wengyuan experimental station (2020), *E2* Guangzhou experimental station (2021), *SD* standard deviation, *CV* Coefficient of variation; h^2^:broad sense heritability

### Genome-wide association analysis

**Fig. 2:**
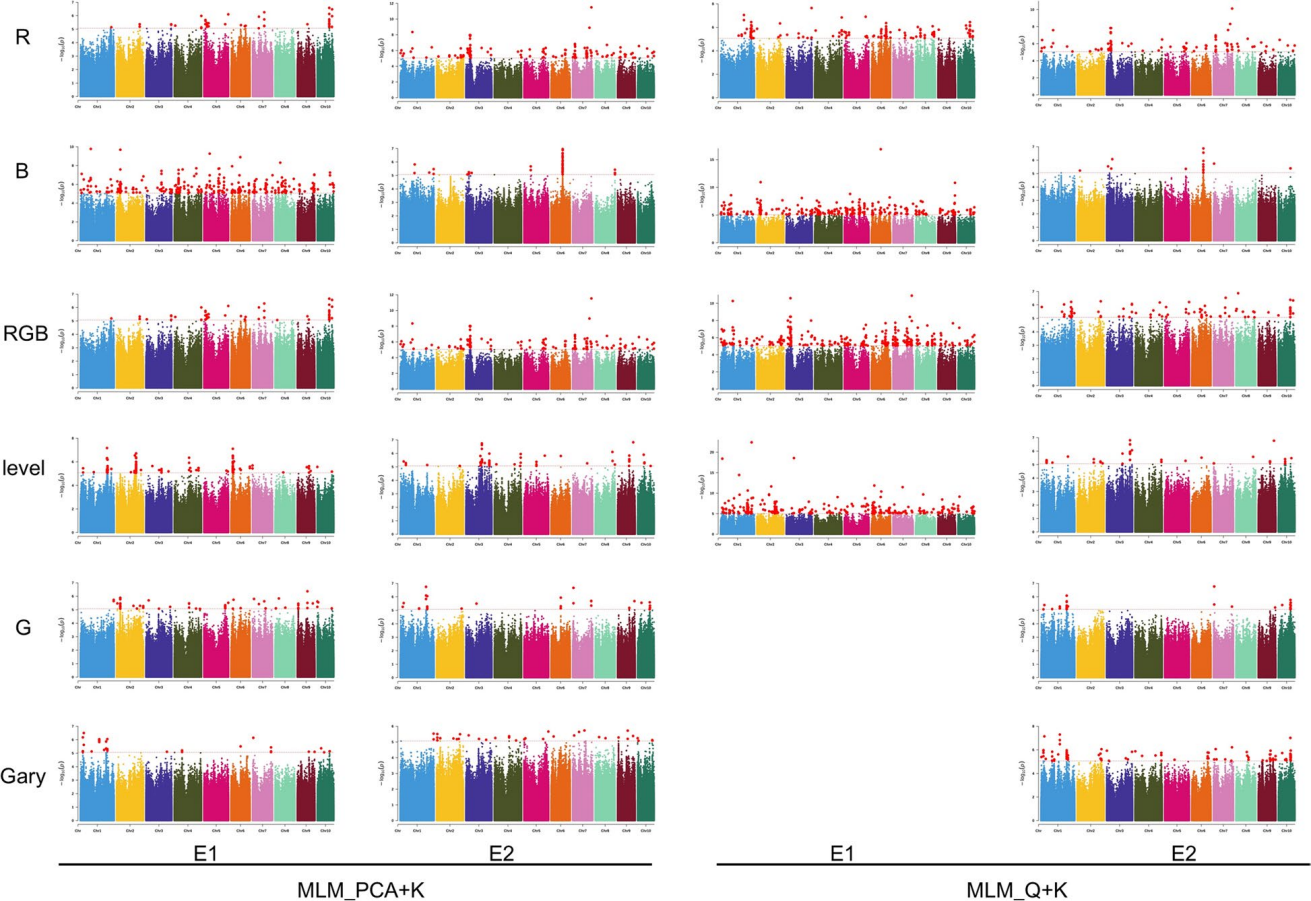
Manhattan-plots for GWAS of 6 kernel colour traits in maize.Two GWAS models for the control of false positive (Q-Q plots). The manhattan plots of two models include MLM_PCA + K (left) and MLM_Q + K (right); E1: wengyuan experimental station (2020); E2: guangzhou experimental station (2021)

**Fig. 3:**
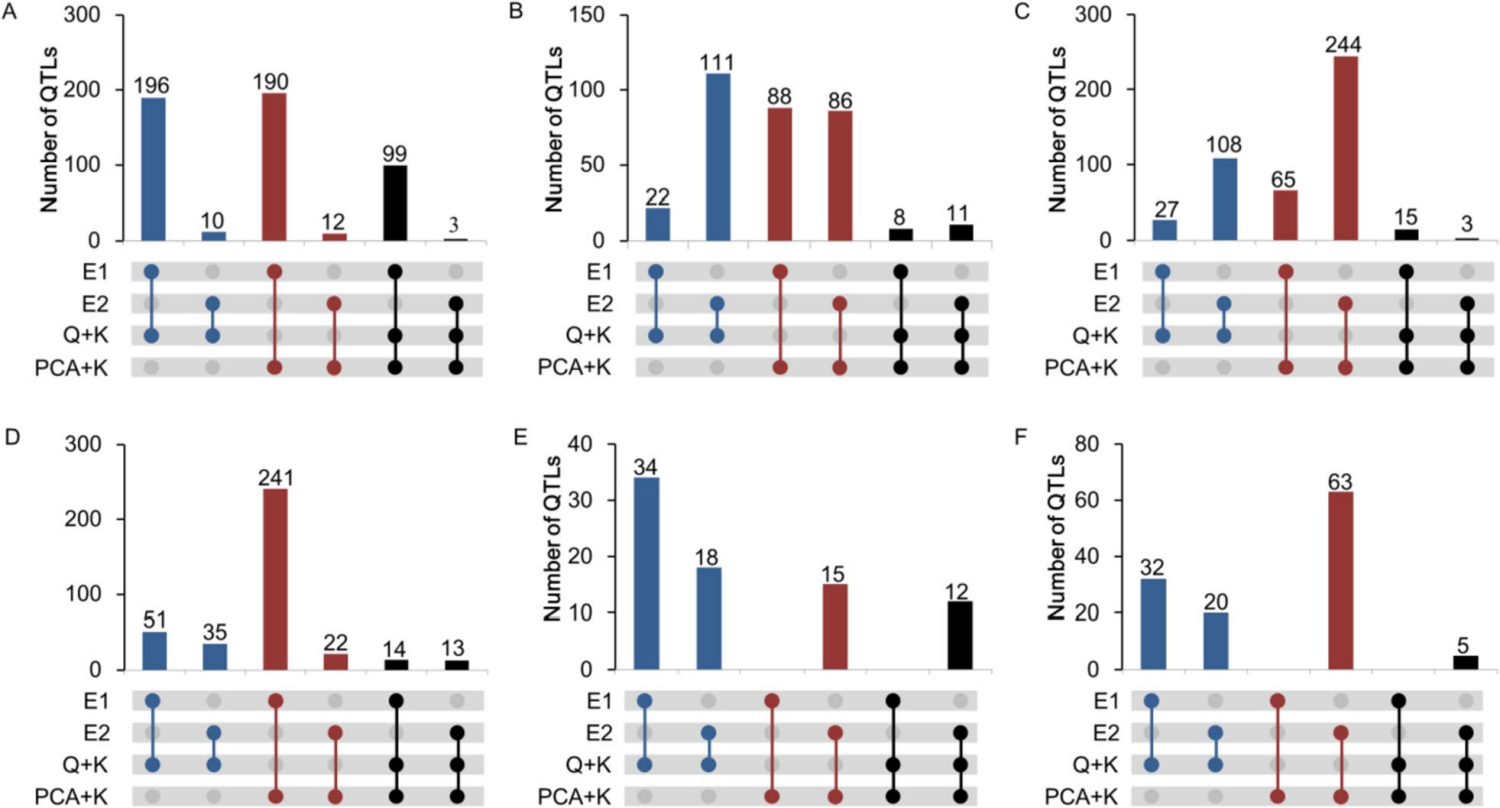
Number of significant QTLs and stable QTLs for the concentration of the 6 kernel colour traits in two environments and GWAS models. **A**, **B**, **C**, **D**, **E** and **F** is R, B, RGB, level, G and Gray, respectively. E1: wengyuan experimental station (2020); E2: guangzhou experimental station (2021). Horizontal bars show the number of QTLs for different environments and methods. The colours of circles corresponding to Horizontal bars indicate the environment in which QTLs was detected and the method applied

GWAS was performed using the Q + K model and PCA + K model in a mixed linear model (MLM), which analysed the 6 colour traits of 244 maize inbred lines across two environments. The QQ plot showed that the model for GWAS was reasonable (Figure S2), and Manhattan plots for each trait are presented in Fig. 2. To combine significant SNPs into QTL intervals The SNPs were in a 50 kb range as a QTL (Table S1, S2, S3 and S4). Under the Q + K model, in total, we identified 877 QTLs significantly associated with kernel colour in the two environments, and 590 QTLs and 440 QTLs were identified in 2020 and 2021, respectively. Among them, 154 QTLs were identified by at least two traits (Fig. 3, Table S4). Under the PCA + K model, we totally identified 475 QTLs significantly associated with kernel colour, 356 QTLs and 304 QTLs in 2020 and 2021, respectively. 163 QTLs of them were identified by at least two traits (Fig. 3, Table S3). A total of 263 QTLs were identified by two models, and 94 were identified by at least two traits. Thirteen QTLs were identified by at least two traits and two environments (Table S5). These loci have important research value and were distributed across the 10 chromosomes.

Two known genes involved in maize carotenoid biosynthesis were detected. A single SNP rs6_85061523 that significant associations with B was detected in the coding region of *Y1*, with a small MAF (0.06) (Table 2), and the R^2^ was 0.29. *Y1* encodes phytoene synthase, which is the key enzyme in the first step of carotenoid biosynthesis and is a typical yellow-and-white gene. The rs3_219867520 that also significant association with B was in the region of *A1 gene*, with a small MAF (0.08), and the R^2^ was 0.1 (Table 2). which encodes bifunctional dihydroflavonol 4-reductase (DFR).

**Table 2:** the carotenoid-related and anthocyanins-related genes within 200 kb of most significant SNP for each trait

Gene ID	Gene	Trait	SNP ID	Chr	Gene position	DD-dd/bp	*p*-value	MAF	R^2^
Zm00001d044122	*A1*	B_1	3_219867520	3	219,866,358	1,162	6.52E-06	0.08	0.1
Zm00001d036345	*y1*	B_1	6_85061523	6	85,064,521	2,998	1.27E-09	0.06	0.29
Zm00001d037382	*cgt1*	RGB_2	6_123785816	6	123,791,605	5,789	2.31E-06	0.07	0.09
Zm00001d045383	*dxs3*	B_1	9_20232174	9	20,239,625	7,451	4.21E-06	0.06	0.18
Zm00001d048469	*hyd5*	Gray_2	9_156874532	9	156,848,938	25,594	3.38E-06	0.12	0.12
Zm00001d012394	*psy2*	RGB_2	8_173494185	8	173,520,611	26,426	1.57E-06	0.12	0.19
Zm00001d034635	*chi1*	R_1	1_298633704	1	298,580,972	52,732	8.31E-06	0.22	0.01
Zm00001d026147	*r1*	B_1	10_139859410	10	139,779,468	79,942	6.77E-06	0.22	0.08
Zm00001d048373	*Wc1/ccd1*	B_1	9_155118340	9	155,234,800	116,460	2.95E-06	0.05	0.15
Zm00001d014914	*A2*	Level_1	5_68147228	5	68,028,581	118,647	4.15E-08	0.07	0.02
Zm00001d038170	*dxs1*	RGB_2	6_150537590	6	150,416,644	120,946	3.76E-06	0.32	0.16
Zm00001d045055	*Bz1*	Level_1	7_19965244	7	20,103,668	138,424	5.05E-06	0.45	0.02
Zm00001d012972	*chi3*	RGB_2;Level_1	5_2392770	5	2,582,304	189,534	1.22E-06	0.06	0.16
Zm00001d012868	*crti3*	R_1	5_1569528	5	1,368,111	201,417	2.42E-06	0.09	0.06

*DD-dd/bp* The distance between gene and SNP, *MAF* Minor-allele frequency

The significant SNP rs9_20232174 was near the previously identified *Dxs3* gene, was approximately 7.4 kb, and had a small MAF (0.06) (Table 2). *Dxs3* encodes a 1-deoxy-D-xylulose 5-phosphate synthase, which catalyses the first and committed step of the MEP pathway [43]. *Cgt1* was located 5.7 kb downstream of rs6_123785816 and MAF (0.07) (Table 2), which encodes c-glucosyl transferase and is a structural gene in the anthocyanin biosynthetic pathway [44]. A SNP located on chromosome 9 (15,687,532) was located 25 kb upstream of *hyd5* and MAF (0.12) (Table 2), which encodes an enzyme with hydroxylase domains and plastid-targeting signals and is involved in carotenoid degradation. *Psy2* was approximately 26 kb away from SNP rs8_173494185 and MAF (0.12) (Table 2), which encodes phytoene synthase and is involved in the carotenoid biosynthesis pathway [45].

In addition, the physical distance between at least 8 QTLs and kernel colour regulation genes is less than 200 kb (Table 2). Three of them are involved in carotenoid biosynthesis: *whitecap1* (*Wc1*) carotenoid cleavage dioxygenase1, which catalyses the cleavage of carotenoids to their corresponding apo-carotenoid products [46]; Dxs1, which catalyses the first and committed step of the MEP pathway [47]; and *Crti3*, which encodes carotenoid isomerase 3 [20]. Five of the genes are involved in the anthocyanin biosynthetic pathway, 4 of which are structural genes: colored1 (R1); *anthocyaninless 2* (A2); *chalcone isomerase 1* (*Chi1*); *chalcone isomerase 3* (*Chi3*) and *Bronze 1* (*Bz1*), which encodes UDP-glucose flavonol glycosyltransferase [4].

### Candidate genes

Based on the B73 reference genome (B73 ref_V4), we obtained 136 candidate genes within 200 kb upstream and downstream of 13 high confidence SNPs, and 95 of them had function annotation (Table S6). Three key candidate genes were selected based on the gene annotation. *Zm00001d048621* encodes an ABC transporter involved in anthocyanin transport; *Zm00001d048626* encodes a cytochrome P450 enzyme; *Zm00001d048623* encodes the MYB transcription factor MYB59.

### The effect of allelic variation

**Fig. 4:**
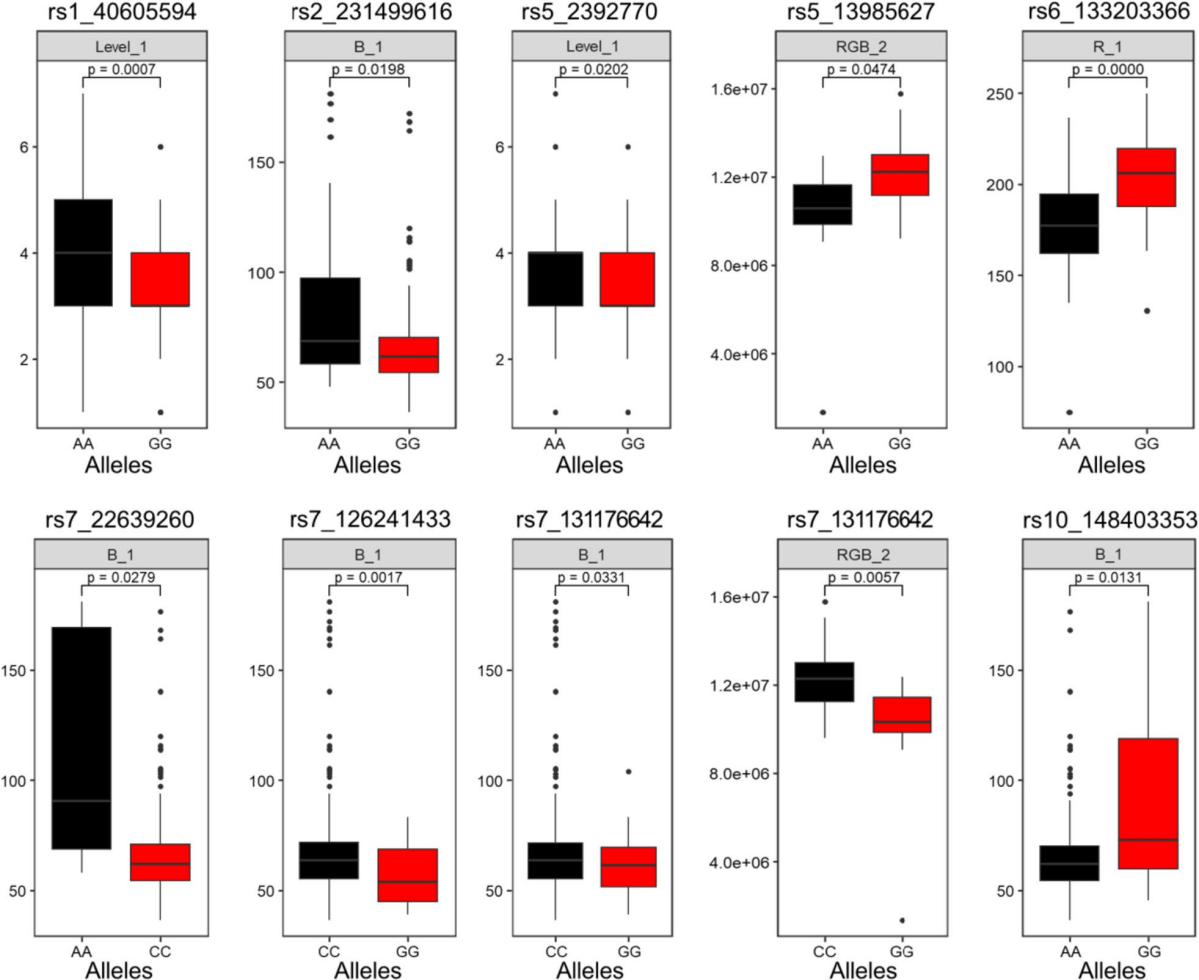
The superior and alternative alleles. _1: wengyuan experimental station (2020); _2: guangzhou experimental station (2021). *P* < 0.05: differences, *P* < 0.01: significant differences, *P* < 0.001: highly significant differences

The R^2^ of 13 SNPs with high credibility ranged from 0.6%-23.2%, and the analysis of the phenotypic data showed that there were significant correlations between the phenotypic data for the 6 kernel colours and each dominant SNP among different allelic variation inbred lines (Fig. 4). For example, AA genotypes at rs1_40605594 sites and rs5_2392770 were largely detected in inbred lines with higher levels, such as yellow or purple kernels rich in anthocyanins and carotenoids. AA genotypes at rs2_231499616 and rs7_22639260 sites were largely concentrated in inbred lines with higher B. Therefore, the SNPs mined in this study have significant effects on maize kernel colour and are important targets for genetic improvement of maize kernel colour.

## Discussion

### Phenotypic analysis of kernel colour

In this study, we performed statistical analysis of the kernel colour phenotype data in two environments and the variation rates of phenotypes were more than 10% higher (Table 1), indicating that the kernel colour of these associated populations was diversity. We also found a certain correlation between two environmental factors (Fig. 1). However, the correlation coefficient was small, which may be due to environmental differences and other factors. A heritability analysis showed that kernel colour had higher heritability (Table 1). This result indicates that kernel colour is mainly regulated by genetic factors, and also influenced by environmental factors [48].

### GWAS model selection analysis

With the rapid development of plant genomics, the development and application of sequencing technology and cost reduction, quantitative trait loci (QTL) and GWAS have been widely used to analyse the genetic basis of plant traits [49]. GWAS is a way to mine genetic variation based on linkage disequilibrium, and there have been many GWAS statistical models [13, 20]. The research shows that the maize GWAS is affected by the community structure and kinship, so choosing the best statistical model to study the relationship between genotypes and traits increases the statistical effect of GWAS [16]. In this study, we used two statistical models, Q + K and PCA + K, and found that the two models could control false positives well (Fig. 2, Figure S2). But because the algorithms vary, Phenotype G and Gary's data in 2020 did not get a reasonable result under the Q + K model, so we analysed the GWAS results of these two models simultaneously.

### Comparative analysis of kernel colour location results

Maize kernel colour is a quantitative trait controlled by multiple genes and has stable heritability. By dividing 2448 inbred lines into 12 levels according to visual kernel colour, and 11 QTLS were identified through linkage analysis, half of which were related to carotenoid biosynthesis genes. Research findings showed that the visual score could be applied to studies of kernel colour [22]. With the same method. Lin et al. (2021) identified a major QTL on chromosome 6 and chromosome 9, and one QTL was *Y1*, which controls yellow and white kernels [23]. Owens identified *Y1* and *Dxs2* by GWAS and explored the relationship between *Dxs3*, *Dmes1*, *LcyE* and *EP1* and kernel colour formation [13]. In this study, both visual scoring and the RGB system were used to evaluate kernel colour, which was taken as phenotypic data, a GWAS was performed for kernel colour-related traits (Fig. 2, Figure S2), and multiple known genes related to kernel colour were identified, such as *Y1*.The rs6_85061523 was in exon 4 of *Y1* and significantly associated with B_1 (Table 2). The *Y1* gene dose effect on endosperm carotenoids was identified in 1940. Sequencing analysis later confirmed that *Y1* encodes phytene synthetase 1 (PSY1), which plays a key role in the formation of phytoene from two molecules of geranylgeranyl pyrophosphate (GGPP) [50]. PSY1 is involved in carotenoid biosynthesis in leaves and endosperm, and its allelic variation to a large extent determines the variation in kernel colour from white to orange [15, 16]. Overexpression of *Y1* can change the colour of the kernel from white to yellow. In addition, *Psy2* is 26 kb downstream of the significant SNP rs8_173494185 (Table 2). *Crti3* encodes a carotenoid isomerase, and the distance from the rs5_1569528 is 201 kb (Table 2). These are all key enzymes in the process by which GGPP produces lycopene [45].

The carotenoid precursor substance GGPP is synthesized by the methylerythritol phosphate (MEP) pathway in higher plants. The key enzyme in the first step of the MEP pathway is 1-deoxy-D-xylulose 5-phosphate synthase (DXS), which is the enzyme with the highest control coefficient in this pathway [51]. In this study, the rs9_20232174 was 7.4 kb away from *Dxs3*, and rs6_150537590 was 121 kb away from *Dxs1* (Table 2). In addition, *hyd5* (*crtRB5*), approximately 25 kb away from rs9_156874532 (Table 2), is involved in hydroxylation reactions downstream of the carotenoid biosynthetic pathway. Carotenoid cleavage dioxygenase 1 (CCD1) is involved in carotenoid degradation. The rs9_155118340 is 25 kb away from *Wc1* (*Ccd1*) [46]. The above findings indicated that the results of this study are highly valuable as a reference.

The anthocyanin synthesis pathway is divided into three stages: the initial reaction of flavonoid metabolism; important reactions of flavonoids; and anthocyanin synthesis [52]. The anthocyanin synthesis pathway is catalysed by a series of enzymes encoded by structural genes, for example, phenyl alanine ammonialyase (PAL) in the first stage; chalcone synthase (CHS), chalcone isomerase (CHI) and flavonoid 3’—hydroxylase (F3’H) in the second stage; and dihydroflavonol4—reductase (DFR) and anthocyanidinaynthase (ANS) in the third stage [27, 28]. In this study, significant signals were detected near *Chi1*, *Chi3* and *A1* (DFR); the rs1_298633704 was located 52 kb downstream of chi1, rs5_2392770 was located 189 kb downstream of *Chi3*, and rs5_68147228 was located 122 kb downstream of *A1* (Table 2).

Anthocyanin skeleton modification is necessary for its maturation, and the most common method of anthocyanin modification is glycosylation, which can enhance the stability and water solubility of anthocyanins. The key enzyme that catalyses this process is UDP-glucose flavonol glycosyl transferase (UFGT) [4, 53]. In this study, a significant SNP rs7_19965244 was found near *Bz1* at a distance of 138 kb (Table 2). In maize, *Bz2* encodes a GST, which helps transport anthocyanins and prevent anthocyanin oxidation, resulting in the bronze colour of kernels [37].

Anthocyanin synthesis structural genes are directly involved in the formation of anthocyanins and their regulation by transcription factors [54]. In this study, the rs3_219867520 was located at the first exon of *R1*, which can activate the expression of *A1* and cause anthocyanin accumulation [55]. The rs10_139859410 located 80 kb downstream of *In1* encodes a bHLH-like inhibitor that increases anthocyanin accumulation in starch [32].

At present, research on the biosynthesis of carotenoids and anthocyanins is fairly clear [7, 8]; however, the mechanism of their regulation of kernel colour formation needs to be studied and explored further. Three key candidate genes were identified in this study. *Zm00001d048623* encodes the MYB transcription factor MYB59. MYB transcription factors are important regulatory factors for the structural genes of the anthocyanin synthesis pathway and are the largest gene family in higher plants [56]. Therefore, *Myb59* may be a key gene that modulates maize kernel colour by regulating anthocyanin synthesis. *Zm00001d048621* encodes an ABC transporter. In maize, *Mrp3* encodes a multidrug resistance-associated protein, an ABC transporter that transports anthocyanins into the vacuole [39]. Thus, we conclude that *Zm00001d048621* is a key gene for anthocyanin transport in maize kernels, which affects kernel colour. *Zm00001d048626* encodes a cytochrome P450 enzyme. In maize, *lut1* encodes CYP97C, and *lut5* encodes CYP97A, which are cytochrome P450-type monooxygenases. LUT1 catalyses the conversion of α-carotene to zeinoxanthin and hydroxylation of zeinoxanthin to yield lutein [20, 22]. CYP97A is an ε-ring carotenoid hydroxylase. Therefore, it is speculated that *Zm00001d048626* encodes a cytochrome P450 enzyme that is involved in the biosynthesis of xanthophylls and regulates kernel colour.

### Identification of superior allelic variation of important loci

There are many superior allelic variations in crop germplasm, such as wild versions or related species, and superior allelic variations of important genetic loci were mined and developed, and new cultivars were bred by molecular assistant selection (MAS) [57]. For example, the diversity of alleles of *LcyE* in maize demonstrates that the favourable allele is more common in tropical lines [19], and the favourable allele for *CrtRB1* is more common in temperate germplasm [58]. In this study, the phenotypic effects of the identified new and pleiotropic loci were analysed, and it was found that the inbred lines carrying different allelic variations had significant differences in phenotype. Moreover, the superior allelic variations of the corresponding loci were identified. rs1_40605594 and rs5_2392770 were significantly associated with the kernel colour level, and selecting A/A superior allelic variation was expected to improve the kernel colour trait (Fig. 4). These results indicate that the superior allelic variations of important loci identified in this study can be used in marker-assisted selection breeding of maize kernel traits for further genetic improvement of crops.

## Conclusions

In summary, we identified 1029 QTLs associated with maize kernel colour by GWAS. Key candidate genes were predicted through functional gene annotation and previous reports, laying the foundation for subsequent gene function verification and providing a reference for analysing the genetic basis of kernel colour and improving the nutritional quality of maize.

## Methods

### Plant materials and field experiments

An association panel was constructed by 244 inbred lines from the laboratory of professor Jinsheng Lai of China Agricultural University containing 3 million SNP markers [59]. These inbred lines were planted in Wengyuan County, Shaoguan City, Guangdong Province (24.35°N, 114.13°E) in 2020 and Haizhu District, Guangzhou City, Guangdong Province (23.10°N, 113.26°E) in 2021. Single row plantings, the row spacing was 65 cm, and intra-row spacing of 25 cm, with the conventional field management and artificial self-pollination. Harvest and dry at post maturity, and then select the consistent maize ears for the further experiments.

### Kernel colour determination

**Fig. 5:**
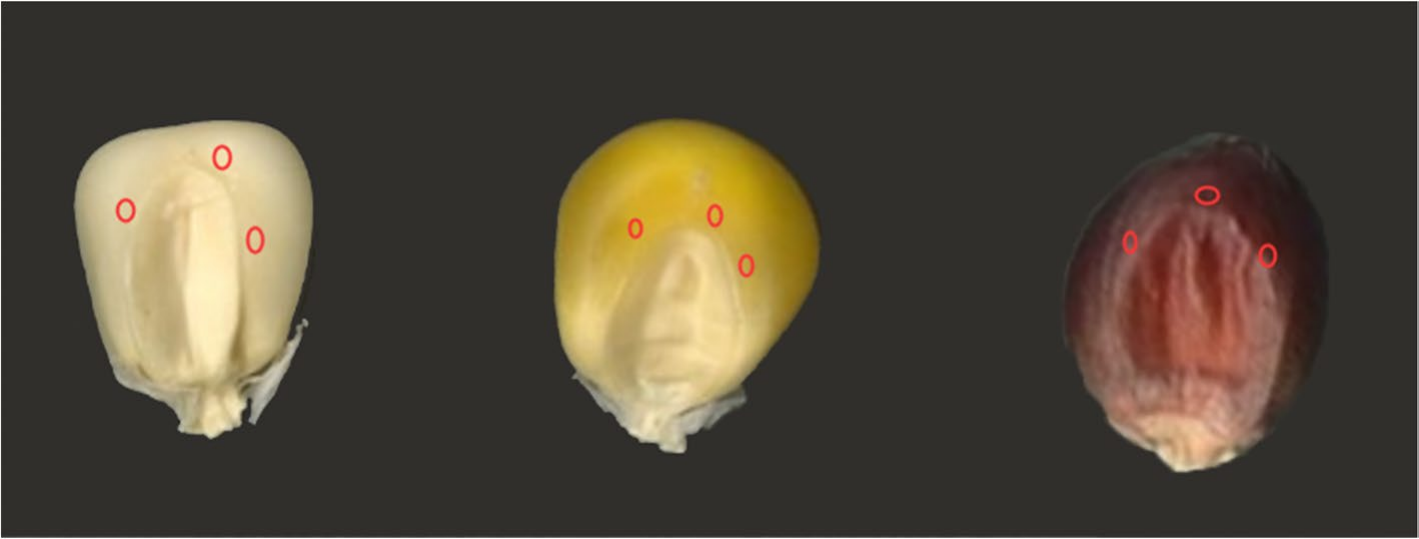
Location diagram of extracting colour values near the maize kernel style. The red circle is where the colour is extracted

**Fig. 6:**
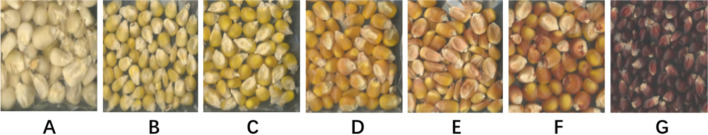
Standardized colour scale representative kernels from the association mapping families. The ordinal colour scale ranges from **A** (lightest) to **F** (darkest), 6 levels

Thirty mature and dry maize kernels with a consistent appearance were selected, and original kernels’ images were captured using EPSON EU-88 scanning devices and EPSON Scan software. The colour values of the top of the endosperm near the style vestige were extracted and calculated based on the image in the RGB colour model, which could get the R, G, B and Gary Value, and RGB = 2562*R + 256*G + B (Fig. 5). In addition, the kernel colour of the inbred lines was graded by visual scoring [22], with the scoring divided into 7 grades, which were used as the visual grade phenotype data for kernel colour (Fig. 6).

The kernel colour data were organized and averaged. The descriptive statistics analysis and data visualization were conducted using IBM SPSS Statistics 25 and R (4.2.2). The Pearson correlation matrix was drawn using the corrplot function and pheatmap package in R statistical analysis program. The Broad-sense heritability (h2) was calculated for kernel colour traits according Nyquist as: h^2^ = δ^2^_G_ / (δ^2^_G_ + δ^2^_E_ / r) where δ^2^_G_ and δ^2^_E_ is genetic variance and residual variance, respectively [60].

### Genome-wide association study

Association analysis for the 6 indexes of colour was conducted via emmax software with a mixed linear model (MLM), taking both the K and Q matrices into account to avoid spurious associations. PLINK was used to calculate the R^2^ of adjacent windows with the parameters of R^2^ > 0.2. A total of 198,910 independent SNPs were ultimately obtained. Then *P* value ≤ 1/198910 (*P* ≤ 1 × 10^–6^) was used as the GWAS significance threshold. Q-Q plot was used to estimate the difference between the observed and predicted *P* values.

To combine significant SNPs into QTL intervals, we combine SNPs within the range of 50 kb as a QTL. If there is only one SNP in the range, we use that as a new starting point and searched forward another 50 kb, the search ends until the distance between two SNPS is larger than 50 kb, SNPs in this range are combined into a QTL, and then the search is repeated with SNPs with distances larger than 50 kb apart as the starting point [61, 62].

### Identification and annotation of candidate genes

According to the linkage disequilibrium analysis of the natural populations, 100 kb was taken as the LD decay distance [63]. All potential candidate genes within 200 kb (100 kb upstream and 100 kb downstream of the lead SNP) of the detected loci were identified. The candidate genes were obtained from the B73 genome reference (version 4) in the MaizeGDB genome browser (https://www.maizegdb.org/). Complementary information was collected from the U.S. National Center for Biotechnology Information (http://www.ncbi.nlm.nih.gov/) and MaizeGDB.

### Analysis of superior allelic variations

On the basis of the results of the GWAS, the most significant SNPs were selected, and the allelic variation effects of these major SNPs were analysed by the R package ofggplot2, ggsignif and ggpubr.

### Supplementary Information

**Additional file 1: Supplemental figure 1.** Frequency distribution of 6 kernel colour traits under two environments. _1: wengyuan experimental station (2020); _2: guangzhou experimental station (2021).** Supplemental figure 2.** Two GWAS models for the control of false positive (Q-Q plots). The X-axis and Y-axis is expected -log_10_(*p*) and observed -log_10_(*p*) of the 6 kernel colour traits in maize; The Q-Q plots of two models include MLM_PCA+K (above) and MLM_Q+K (below); E1: wengyuan experimental station (2020); E2: guangzhou experimental station (2021).

**Additional file 2: Table S1.** SNPs identified by PCA+K models in two environments. **Table S2.** SNPs identified by Q+K models in two environments. **Table S3.** QTLs identified by PCA+K models in two environments. **Table S4.** QTLs identified by Q+K models in two environments. **Table S5.** The list of significant SNPs identified by at least two traits and two environments. **Table S6.** The information of candidate genes.

## Data Availability

All data generated or analyzed during this study are included in this article and its supplementary information files or are available from the corresponding author on reasonable request.
