# Supplementary material for: Genome-wide association study of kernel colour traits and mining of elite alleles from the major loci in maize
Source: BMC Plant Biol. 2024 Jan 3;24:25. doi: 10.1186/s12870-023-04662-5 (PMC10763400; doi:10.1186/s12870-023-04662-5)
Supplement: Supplementary file 1 — Additional file 1: Supplemental figure 1. Frequency distribution of 6 kernel colour traits under two environments. _1: wengyuan experimental station (2020); _2: guangzhou experimental station (2021). Supplemental figure 2. Two GWAS models for the control of false positive (Q-Q plots). The X-axis and Y-axis is expected -log10(p) and observed -log10(p) of the 6 kernel colour traits in maize; The Q-Q plots of two models include MLM_PCA+K (above) and MLM_Q+K (below); E1: wengyuan experimental station (2020); E2: guangzhou experimental station (2021). [file 12870_2023_4662_MOESM1_ESM.docx]

**Supplementary data**


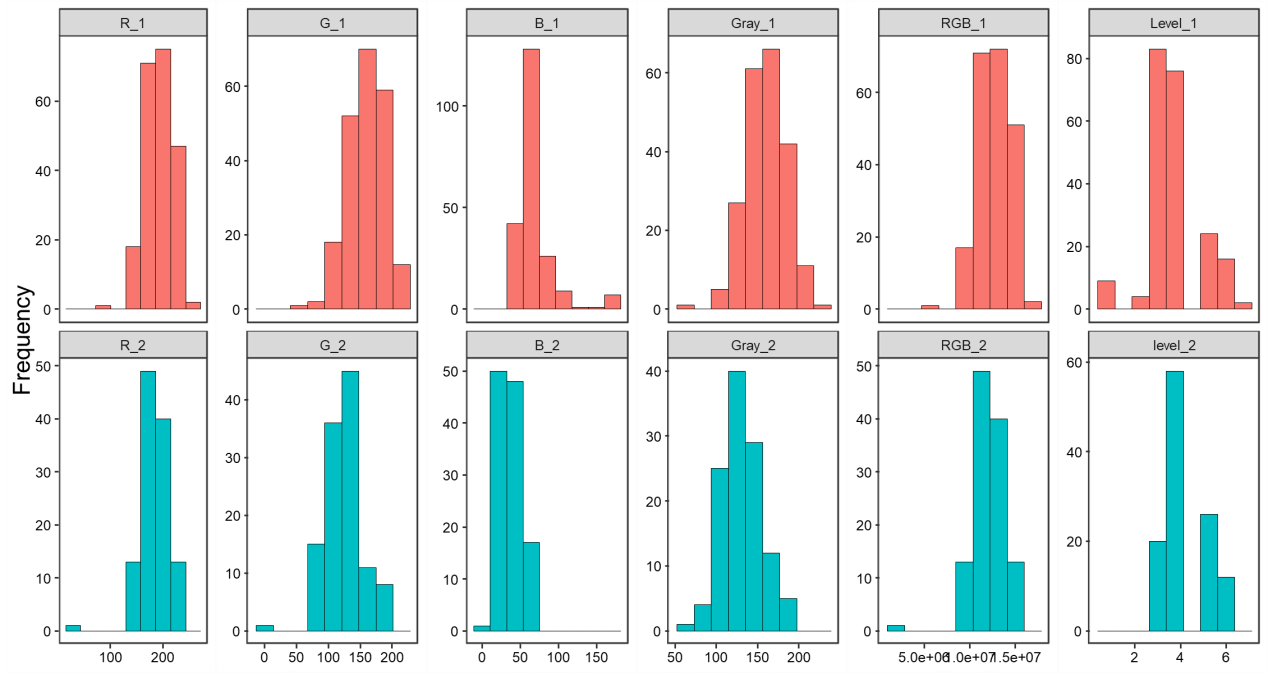


**Supplemental figure 1 Frequency distribution of 6 kernel colour traits under two environments.** _1: wengyuan experimental station (2020); _2: guangzhou experimental station (2021).


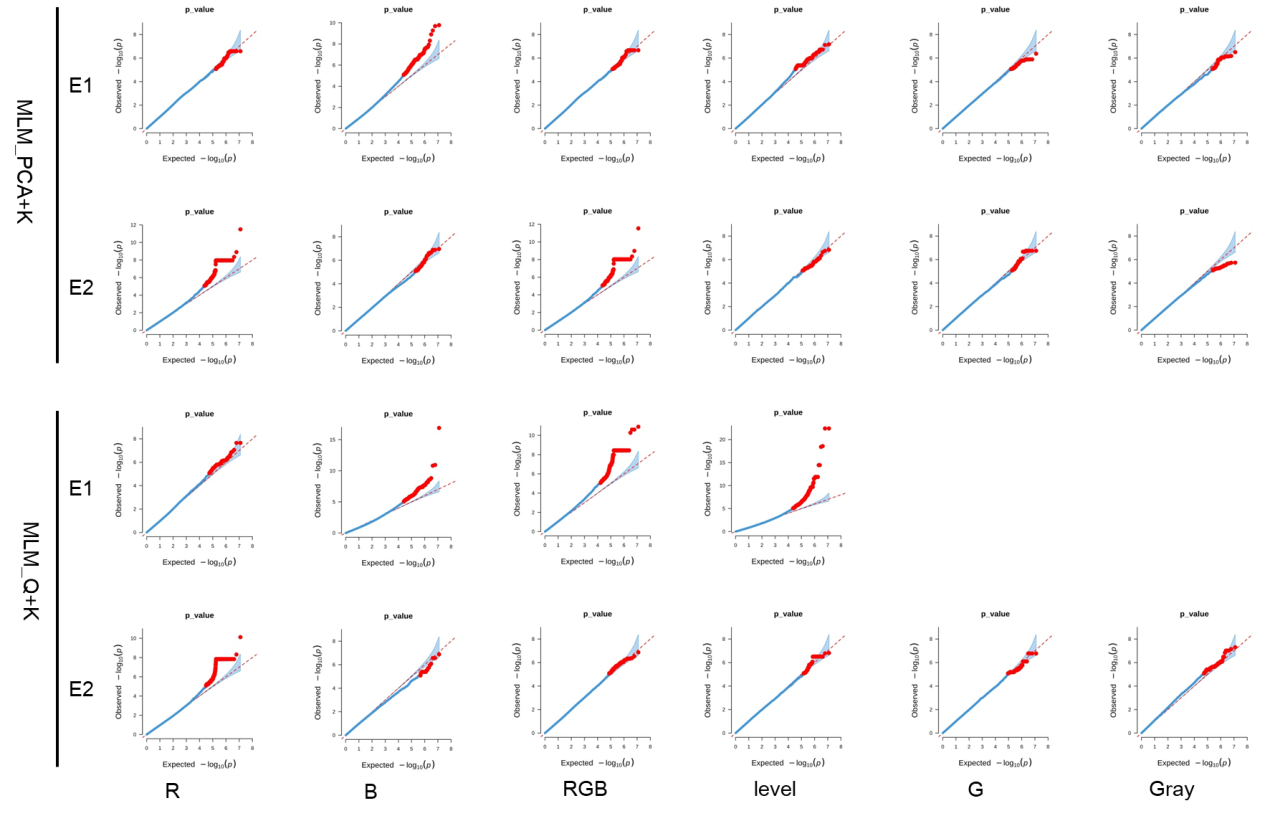


**Supplemental figure 2 Two GWAS models for the control of false positive (Q-Q plots).** The X-axis and Y-axis is expected -log_10_(*p*) and observed -log_10_(*p*) of the 6 kernel colour traits in maize; The Q-Q plots of two models include MLM_PCA+K (above) and MLM_Q+K (below); E1: wengyuan experimental station (2020); E2: guangzhou experimental station (2021).
